# Appraising the need for care in alzheimer’s disease

**DOI:** 10.1186/1471-244X-13-73

**Published:** 2013-03-04

**Authors:** Claudia Schiffczyk, Barbara Romero, Christina Jonas, Constanze Lahmeyer, Friedemann Müller, Matthias W Riepe

**Affiliations:** 1 Department of Psychiatry and Psychotherapy II, Mental Health & Old Age Psychiatry, Ulm University, Ulm, Germany; 2 Alzheimer Therapiezentrum Bad Aibling, Bad Aibling, Germany; 3 Freelance Psychologist, Berlin, Germany

**Keywords:** Dementia, Care level, Nursing care insurance, Caregiver

## Abstract

**Background:**

Increasing incidences of dementia necessitate the improvement of supportive measures for patients suffering from this disease and their proxies. Clinicians without psychiatric backgrounds and others involved in appraising the supportive needs of dementia patients, such as those who allocate nursing insurance, base their appraisals on the ability of patients to perform basic and instrumental activities of daily living (B-ADL, iADL). Our aim was to investigate whether a reduced ability of the patient to perform ADL is sufficient to adequately assess the supportive needs of family caregivers.

**Methods:**

Cross-sectional baseline data were obtained from dementia patients and their proxies in the context of a nationwide prospective cohort study on non-pharmacological treatment of dementia. To our knowledge, the present study is the first country-wide study to assess patients and proxies in their domestic surroundings (e.g. Mini-Mental State Examination (MMSE) Behave-AD, B-ADL and iADL for patients; Quality of Life (QOL) and depression of the proxy).

**Results:**

Logistic and linear regression analysis show that the allocation of nursing care allowance provided by German mandatory nursing insurance is associated with scores on the B-ADL- and iADL scales, but not with the severity of behavioural symptoms or the supportive time the proxies spend on caring. However, the severity of cognitive and non-cognitive symptoms of dementia patients, correlate with each other and both parameters correlate with the time the proxy spends on caring. The time spent on caring is associated with an increase in depression and a reduction in the quality of life of the proxy.

**Conclusions:**

Basic and instrumental activities of daily living do not sufficiently reflect the perceived burden of care experienced by the proxy who has to cope with the imposition of the dementia patients’ behavioural symptoms. When allocating nursing care, patients’ behavioural symptoms should also be taken into consideration, because depressive symptoms of proxies are linked to non-cognitive symptoms in dementia patients. To provide better health care, it is necessary to identify and treat psychiatric symptoms in proxies who care for dementia patients as early as possible.

## Background

Because of demographic changes, the prevalence and incidence of dementia patients and the need for more caregivers will increase in the future [1]. The health care system will therefore be challenged to provide cost-effective treatment while being constrained by limited resources. Approximately 1.2 million people in Germany suffer from dementia and it has been estimated that this number will double in the future [2].

Alzheimer’s disease (AD) is characterized by both cognitive and neuropsychiatric symptoms. ‘Cognitive symptoms’ include aspects such as attention span deficit, memory impairment, visual-perceptive and orientation deficits, apraxia, and agnosia. ‘Non-cognitive’ symptoms or ‘behavioural and psychological symptoms of dementia (BPSD)’, comprise a broad spectrum of symptoms such as depression, agitation, delusions, irritability, and stereotypic motor behaviour [3]. The chronological appearance of cognitive symptoms is somewhat predictable in the progression of AD, whereas non-cognitive symptoms present in a heterogeneous fashion and appear inconsistent. Non-cognitive symptoms can appear, fluctuate in their intensity and sometimes fade without treatment [4]. These cognitive and behavioural symptoms inevitably influence the occurrence and worsen the impairments of activities of daily living [5]. Patients increasingly need help in tasks such as dressing, preparing meals, shopping for groceries, or taking care of personal hygiene. These BPSD, although varying in frequency and severity [6], negatively increase the burden experienced by proxies who care for dementia patients [7]. In Germany, about two thirds of patients with dementia live in private households and are cared for by their proxies [8]. In the early and moderate stages of dementia, symptoms and disease-related disturbances are compensated for by caregivers, especially when patients suffer from anosognosia: a lack of awareness of their deficits [9]. The general health risk of caregivers is therefore increased in proxies who care for such dementia patients [10-13].

In Germany, consultants from the medical service of mandatory health insurance (Medizinischer Dienst der Krankenversicherung (MDK)) evaluate the patients’ needs for support and decide on allowances for nursing care after visiting them. These consultants rely on their personal appraisal skills and standardized questions. The evaluation predominantly focuses on the ability of the patient to perform activities of daily living (ADLs) such as personal hygiene, mobility, food intake and household chores.

Even in the early stages of disease, patients’ decision making and awareness of their deficits is often impaired [14,15]. In fact, a substantial number of patients with early AD erect a façade and deny having problems when asked about their deficits [14,15]. When being evaluated, they try to perform tasks as capably as possible and often perform better than they would under normal circumstances, thus making evaluation even harder. Cognitive impairment is the predominant symptom in the early stages of dementia. With the progression of the disease, behavioural symptoms increase in frequency and severity [16], which poses an increased burden on family and caregivers [17,18]. Behavioural symptoms of patients might, to a certain extent, be accountable for the premature institutionalization of patients with AD, which in turn imposes challenges on the health care system. In the long run, costs for hospital admissions or premature transfers to nursing institutions are higher than for providing preventive support for caring proxies before the dementia patient’s symptoms exacerbate. Behavioural symptoms are not considered in the evaluation by medical consultants who decide upon the allocation of allowances from nursing care insurance. Our assessments were equivalent to the evaluation of the medical consultants: patients and proxies were interviewed in their domestic surroundings by specifically trained and experienced research assistants. It was the goal of the present study to investigate whether restrictions on the appraisal of ADLs sufficiently captured the support needs of family caregivers.

## Methods

The study was performed according to the institutional guidelines set down by the Ethics Committee at Ulm University and the principles laid out in the Declaration of Helsinki. Informed written consent was obtained by patients and proxies.

### Patients and caregivers

We analysed cross-sectional baseline data in the context of a prospective cohort study from patient- and proxy-dyads applying for, or asking for, information about a short-term, in-patient treatment at the Alzheimer Therapy Center (Alzheimer Therapiezentrum, ATZ) in Bad Aibling. The duration of this short-term, in-patient treatment was three to four weeks. Initial contact and screening regarding the eligibility for inclusion into this study was made via a telephone call. Criteria for inclusion in this naturalistic study was extended to people with dementia of mixed or Alzheimer’s type who had been diagnosed by either a general practitioner or a neurologist/psychiatrist according to their routine diagnostic procedures. Considering that no predefined standardized set of diagnostic criteria had been used by the practitioners, analysis of subgroups of patients with “pure” Alzheimer’s disease and mixed dementia were precluded. Additionally, only patients living in the same household with their caregiver were included in the study; most caregivers were spouses of the patients. This analysis comprises baseline assessments between September 2008 and June 2010 of all patients that had a Mini-Mental Status Examination (MMSE) of 3 and above, and were able to complete the Geriatric Depression Scale (GDS).

All interviews took place in the patient’s domestic surrounding which included their family. After explaining the aim of the study and obtaining informed consent by both the patient and the proxy, assessments were then carried out by specially trained research assistants. Patients and proxies were interviewed separately to minimize bias and avoid any mutual influence when giving their responses.

### Assessments

#### Mini-mental status examination (MMSE)

The MMSE [19] is the most commonly used instrument to gauge the severity of dementia by assessing cognitive functions. It comprises tests on orientation, registration, short-term memory, language use, comprehension, and basic motor skills. The score ranges from 0–30. Patients are considered to be in a mild stage of the disease when scoring 20 points or above; in a moderate stage when scoring between 10 and 19; and in a severe stage when scoring 9 or less.

#### Behavioural pathology in the Alzheimer’s disease rating scale (Behave-AD)

The Behave-AD [20] is a clinical rating instrument to characterize the phenomenology of behavioural symptoms. It comprises 25 items, all of which are answered by the proxy in the following seven categories: delusions, hallucinations, motor disturbances, aggression, circadian rhythm, affective symptoms and panic disorders/phobias.

#### Geriatric depression scale (GDS)

The Geriatric Depression scale [21] is a 15-item questionnaire to assess symptoms of depression and has been validated for both cognitively unimpaired and demented elderly [22,23]. A score of 5 or above indicates a clinically relevant depression level [24]. While the scale was designed to assess depressive symptoms in patients older than 60 years of age, one of the limitations of this study is that a minority of subjects younger than this also completed this scale.

#### Activities of daily living (Bayer–ADL)

The Bayer-ADL scale [25] is used to assess deficits in the performance of patients’ everyday activities. It comprises 25 items, all of which are answered by the proxy. Ratings are made on a 10-point, Likert-type scale. Lower scores indicate less functional impairment.

#### Instrumental activities of daily living (iADL)

This iALD scale [26] is used to assess the deficits in the performance of the patients’ everyday activities. It comprises eight items, with lower scores indicating a deteriorating performance in activities.

#### Quality of life in Alzheimer’s disease (QOL-AD)

The QOL-AD [27] is a 13 item questionnaire designed to appraise the patient’s quality of life. It has also been used to appraise the quality of life of healthy elderly controls [28]. The QOL-AD covers the following domains: physical health, energy, mood, living situation, memory, family, marriage, friends, household chores, fun, money, self and life as a whole and answers are given on a 1–4 point range: 1 is poor and 4 is excellent. Total scores range from 13–52 with higher scores indicating a better quality of life. In this study the QOL-AD was administered twice: once to the caregiver and once as a substituted judgement of the patient’s quality of life: the caregiver was asked to rate the QOL of the patient as the patient would do, if he or she was still capable to do so.

#### Resource utilization in dementia – the short version (RUD lite)

The RUD Lite [29] is a short version of the RUD structured interview that asks proxies about the time spent in taking care of their patient. It comprises questions about the total hours spent by the proxy in supporting the patients to complete tasks, including: ADLs such as using the bathroom, feeding, toileting and showering/taking baths; iADLs such as shopping for groceries, preparing meals, undertaking household chores, doing the laundry; and supervising the patient.

#### Nursing care insurance

In this study we differentiated between whether an allowance was given for nursing care or not. Further discrimination regarding the degree of allowance was not analysed as many of the proxies were unable to reliably report on this when questioned.

### Data analysis

All statistical analyses for the investigation of group differences were carried out using the statistics program SPSS (SPSS 15.0 for Windows, Chicago, Ill., 2001). The normality of distribution was tested with the Kolmogorov-Smirnov Test. MMSE, word fluency of the patient and the GDS of the proxy were normally distributed, while all other variables were not. Correlation coefficients were calculated using Spearman’s rho.

The allocation of nursing care in the present study was a dichotomous variable. We therefore used a logistic regression model to predict the variables influencing this factor.

All other variables were metric, and we therefore used a linear regression model to predict which ones influenced the depressive syndrome of the proxy, the quality of life of the proxy, and the subjective burden experienced by the proxy.

Confidence intervals of 95% were calculated and p-values smaller than 0.05 were considered to be significant.

## Results

From an initial cohort of 212 patient-proxy dyads, 10 were excluded due to a false diagnosis (n = 9) or death (n = 1) and 8 more were not able to fill out the GDS. Data from the remaining 194 patient-proxy dyads were analysed in this study. At baseline, the patients were diagnosed with either AD or mixed dementia with a mean age of 73.0 ± 7.1 years, an age range of 52–89 years and 73.3% were male. The proxies’ mean age was 69.0 ± 7.7 years, their age range was 43–90 years and 72.2% were female. MMSE scores ranged from 3–28, with a mean of 17.2 ± 6.8. Demographic variables are shown in Table 1.

**Table 1:** Demographic data of patients and proxies

	**Proxies**			**Patients**		
	**All**	**Males**	**Females**	**All**	**Males**	**Females**
Age (years)						
N	194	54	140	194	137	57
n < 60	28	1	27	8	2	6
min/max	43/90	54/90	43/84	52/89	55/89	52/86
Mean	69.0	72.2	67.7	73.0	73.3	72.4
SD	7.7	6.6	7.7	7.1	7.1	7.1
MMSE (score)						
min/max				3/28	3/28	3/28
Mean				17.2	17.1	17.4
SD				6.8	6.8	6.7

**Figure 1:**
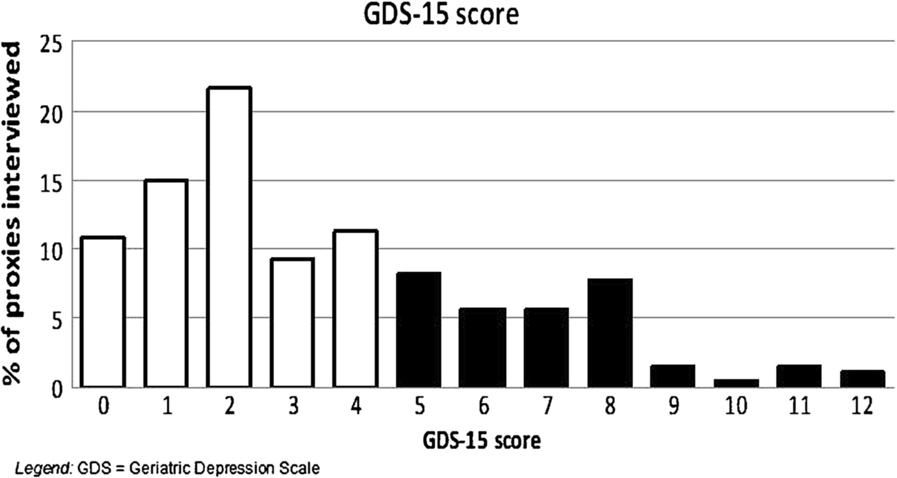
**Depression levels of proxies caring for dementia patients.** A score < 5 is considered normal (white bars) while a score of 5 or above is considered to reflect clinically relevant depressive symptoms (black bars).

About one third (n = 61, 26.2%) of the caregivers suffered from clinically relevant depressive symptoms according to the previously published criteria associated with the use of the GDS (Figure 1).

In AD patients, the severity of cognitive (MMSE) and non-cognitive symptoms (Behave-AD) correlated with one another (rho = −0.310; p < 0.001). The severity of the patients’ cognitive symptoms did not correlate with the depression of the proxies (GDS: rho = −0.128, p = 0.075) or their self-assessed quality of life (QOL -AD; rho = 0.085, p = 0.238). However, there is an association between the depression of the proxies and their quality of life with the non-cognitive symptoms of the patient (GDS: rho = 0.330, p < 0.000; QOL -AD: rho = −0.275, p < 0.000). Mean time of the time spent on care and supervision are displayed in Table 2. The time the proxy spends on caregiving correlates with the extent of both cognitive and non-cognitive deficits and functional deficits (Table 3).

**Table 2:** Mean time spent on care and supervision

	**Mean (in h )**	**SD**	**Range**
RUD – time ADL	1.49	1.58	0 – 8
RUD – time iADL	2.06	1.51	0 – 8
RUD – time supervision	18.41	7.94	0 – 24

**Table 3:** Correlation (Spearman rho) between the time the proxy spent on care (Rud Lite) and the symptoms of the patient (**: p < 0.001; *: p = 0.017)

	**RUD – time ADL**	**RUD - time iADL**	**RUD – time supervision**
B-ADL	0.721**	0.423**	0.646**
iADL	−0.722**	−0.381**	−0.551**
Behave-AD	0.399**	0.248**	0.412**
MMSE	−0.532**	−0.171*	−0.458**

A logistic regression analysis (including health care allowances, MMSE, Behave-AD, B-ADL, iADL, RUD – time spent on ADL, RUD – time spend on iADL, RUD – time spent on supervision) shows that the allocation of a nursing care allowance is associated with scores of the B-ADL- and iADL-scales, but not with the severity of behavioural symptoms or with the time the caregiver spends on taking care of the patient. These results remain unchanged, even after limiting analysis to mild and moderate stages of the disease (MMSE ± 10) (Table 4).

**Table 4:** Binary logistic regression analysis with the allowance of nursing care as the dependent variable

**Predicting variables**	**B**	**SE**	**Wald**	**p-value**	**OR (95% CI)**
Total (MMSE >3)					
B-ADL	−.347	.137	6.434	.011	.707 (0.541 – 0.924)
iADL	.440	.147	8.911	.003	1.552 (1.163 – 2.070)
Mild to moderate dementia (MMSE > 10)					
B-ADL	−.331	.141	5.488	.019	.718 (0.544 – 0.947)
iADL	.442	.155	8.135	.004	1.555 (1.148 – 2.107)

Legend: B = Regression Coefficient, SE = Standard Error of B, OR = Odds Ratio, CI = Confidence Interval.

Regression analysis on the depression of the proxies as a dependent variable and symptoms of the patients as an independent variable, show both impaired ADLs of the patient and behavioural symptoms of the patient to be associated with the depression of their proxies. Subjective burden experienced by the proxies is associated with non-cognitive symptoms of the patients, their ability to perform ADLs, and the self-assessed time the proxy spends on supporting the patient in ADLs and iADLs (Table 5).

**Table 5:** Linear regression analysis with regard to depression levels, quality of life of the proxy and subjective burden experienced by the proxy as dependent variables

**Predicting variables**	**UB**	**SE**	**SB**	**t**	**p-value**	**95% CI**
Depression of proxy (GDS)						
Behave-AD	.120	.044	.205	2.711	.007**	0.27 – 0.205
Bayer-ADL	.332	.154	.264	2.165	.032**	−0.007 – 0.645
iADl	−.021	.164	−.015	−.127	.899	−0.341 – 0.324
MMSE	.032	.032	.077	.998	.320	−0.026 – 0.106
RUD time on ADL	.087	.160	.049	.542	.589	−0.229 – 0.403
RUD time on iADL	−.121	.142	−.065	−.849	.397	−0.400 – 0.159
RUD time on supervision	.015	.030	.045	.503	.616	−0.044 – 0.075
Constant	−.145	1.606		−.090	.928	−3.605 – 2.901
Quality of life of the Proxy (QOL-AD)						
Behave-AD	−.138	.085	−.127	−1.618	.107	−0.305 – 0.030
B-ADL	−.688	.293	−.298	−2.351	.020**	−1.266 – − 0.111
iADl	−.267	.313	−.108	−.855	.394	− 0.884 – 0.350
MMSE	−.057	.062	−.075	−.928	.355	−0.179 – 0.064
RUD time on ADL	.199	.306	.061	.651	.516	−0.404 – 0.803
RUD time on iADL	.279	.271	.081	1.032	.303	−0.255 – 0.813
RUD time on supervision	.049	.058	.079	.851	.396	−0.065 – 0.163
Constant	44.327	3.068		0.000	14.447	38.275 – 50.380
Burden of the proxy (Behave-AD)						
Behave-AD	.063	.014	.329	4.391	.000**	0.035 – 0.091
B-ADL	.124	.052	.303	2.379	.018**	0.021 – 0.228
iADl	.067	.053	.152	1.247	.214	−0.039 – 0.172
MMSE	.000	.011	.000	−.005	.996	− 0. 021 – 0.021
RUD time on ADL	−.101	.051	−.174	−1.986	.048**	− 0.202 – 0.001
RUD time on iADL	.105	.045	.173	2.338	.020**	− 0.016 – 0.194
RUD time on supervision	−.003	.010	−.030	−.343	.732	−0.022 – 0.016
Constant	.542	.523		1.037	.301	−0.489 – 1.573

Legend: UB = Unstandardized B, SE = Standard Error of B, SB = Standardised B, CI = Confidence Interval.

## Discussion

The present nationwide study was conducted within the domestic surroundings of patients and their proxies, therefore enabling assessments of the symptoms and the burden of both the patients and the proxies to be undertaken in their everyday environment. Analogous to studies carried out in other settings [30], the results of our study show that the impact of dementia on caregivers is underestimated when only the functional level is addressed by assessing basic and instrumental activities of daily living, without considering possible depressive symptoms of the proxies.

In the present study, around one third of the patients suffered from clinically relevant depressive symptoms. This result supports the estimation made by neurologists in a recently published survey [31]. It is known that depression negatively affects the ability of the proxy to provide good patient-centred care and at the same time this state affects their own overall health. Studies have shown that caring for a person with dementia is associated particularly with depression [12], the proxies’ own health related issues [12] and the mortality rate of the caring proxy [10].

The revision of the 1996 long-term care insurance in Germany states that the estimation of the need for care of dementia patients should be based on the ability of the patient to perform certain activities in daily living. Using these standards, the benchmark of this evaluation is exclusively the ability to carry out or perform such activities, but not the severity or nature of the disease at hand. Results of the present study suggest that the burden or the strain the patients and the proxies suffer from, are only partially captured when only these standards are considered. Based on the present results, non-cognitive symptoms, which are very prevalent in AD, are not sufficiently considered when nursing allowances are assigned. Previous studies have already demonstrated that neuropsychiatric symptoms account for the burden experienced by the caregiver and are a crucial reason for the institutionalization of dementia patients [32,33]. Therefore, the assessment of basic and instrumental activities of daily living only partially reflect the need for help, and any assessment should be extended by taking dementia-specific neuropsychiatric symptoms into account.

## Conclusions

In summary, the need to support family caregivers of dementia patients should not be solely appraised on the grounds of the patient’s ability to perform activities of daily living. Consideration should also be given to the presence of any neuropsychiatric and behavioural symptoms. In addition, when treating dementia symptoms in patients, clinicians need to take into account the possibility that caregivers may also need treatment for their depressive symptoms.

## Competing interests

The present study was funded by the German Federal Ministry of Health (MNG-LTDEMENZ_04_61). The sponsor did not influence the design of the study, the analysis of the data, or the drafting of the manuscript. CJ, CL and CS report no conflict of interest. BR, FM and MWR have received grants or funding and honorarium from companies selling or developing medical products for use in patients with Alzheimer’s disease. None of the authors report a personal or financial conflict of interest.

## Authors’ contributions

CJ, CL, and CS were involved in the acquisition of data, data analysis, and the drafting and revising of the manuscript. BR, FM and MWR were involved in designing the study, interpreting the data, and the drafting and revising of the manuscript. All authors approved the final version of the manuscript.

## Pre-publication history

The pre-publication history for this paper can be accessed here:

http://www.biomedcentral.com/1471-244X/13/73/prepub
